# Lymphoblastoid cell lines from Diamond Blackfan anaemia patients exhibit a full ribosomal stress phenotype that is rescued by gene therapy

**DOI:** 10.1038/s41598-017-12307-5

**Published:** 2017-09-20

**Authors:** Anna Aspesi, Valentina Monteleone, Marta Betti, Chiara Actis, Giulia Morleo, Marika Sculco, Simonetta Guarrera, Marcin W. Wlodarski, Ugo Ramenghi, Claudio Santoro, Steven R. Ellis, Fabrizio Loreni, Antonia Follenzi, Irma Dianzani

**Affiliations:** 1 Department of Health Sciences, Università del Piemonte Orientale, Novara, Italy; 2 Department of Biology, University of Rome Tor Vergata, Roma, Italy; 3 Department of Medical Sciences, University of Torino, and Human Genetics Foundation (HuGeF), Torino, Italy; 4 Department of Paediatrics and Adolescent Medicine, Division of Paediatric Hematology and Oncology, Medical Center, Faculty of Medicine, University of Freiburg, Freiburg, Germany; 5 Department of Public Health and Paediatric Sciences, University of Torino, Torino, Italy; 6 Department of Biochemistry and Molecular Genetics, University of Louisville, Louisville, KY USA

## Abstract

Diamond Blackfan anaemia (DBA) is a congenital bone marrow failure syndrome characterised by selective red cell hypoplasia. DBA is most often due to heterozygous mutations in ribosomal protein (RP) genes that lead to defects in ribosome biogenesis and function and result in ribosomal stress and p53 activation. The molecular mechanisms underlying this pathology are still poorly understood and studies on patient erythroid cells are hampered by their paucity. Here we report that RP-mutated lymphoblastoid cell lines (LCLs) established from DBA patients show defective rRNA processing and ribosomal stress features such as reduced proliferation, decreased protein synthesis, and activation of p53 and its target p21. These phenotypic alterations were corrected by gene complementation. Our data indicate that DBA LCLs could be a useful model for molecular and pharmacological investigations.

## Introduction

Diamond Blackfan anaemia (DBA, OMIM #105650) is a rare congenital hypoplasia of erythroid progenitors characterised by normochromic macrocytic anaemia with normal leukocytes and platelets, and, in one third of cases, congenital malformations^1,2^. DBA is associated with an increased risk for myelodysplastic syndrome (MDS), acute myeloid leukaemia (AML), and solid tumours^3^. Patients are treated with steroids or chronic transfusions; the only curative therapy for the haematologic manifestations of DBA is haematopoietic stem cell transplantation. Most cases of DBA are caused by heterozygous mutations in ribosomal protein (RP) genes that cause haploinsufficiency and impairment of ribosome biogenesis and function. So far, mutations in 19 RP genes (*RPS19, RPS24, RPS17, RPL35A, RPL5, RPL11, RPS7, RPS10, RPS26, RPL26, RPL15, RPL31, RPS29, RPS28, RPL27, RPS27, RPS15A, RPL35, RPL18*) have been identified in DBA patients, including deletions, missense, nonsense and splice mutations^4–17^. *RPS19* is the most frequently mutated gene and accounts for 25% of cases^18^. Mutations in RPs of the small (RPS) or large (RPL) ribosomal subunits affect various steps of ribosomal RNA (rRNA) maturation, resulting in the accumulation of specific rRNA precursors^12,18,19^. Ribosomal stress, also named nucleolar stress, induced by RP deficiency triggers p53 stabilisation and activation^20^, as well as p53-independent pathways^21^ probably leading to impaired proliferation and/or apoptosis of the erythroid progenitors in the bone marrow^22,23^. Since patients’ erythroid progenitors are not easily accessible for research purposes, several animal and cellular models of DBA have been developed with the aim of elucidating the molecular mechanisms of the disease. Disruption of one *Rps19* allele in mice does not recapitulate DBA, possibly because of some unknown compensation mechanism^24^, while zebrafish models show both haematopoietic and developmental anomalies, resembling DBA^25–27^. Recently, induced pluripotent stem cells (iPSCs) were generated from fibroblasts of DBA patients carrying mutations in *RPS19* or *RPL5* and their use has been proposed for drug screening^28,29^.

Lymphoblastoid cell lines (LCLs) established from DBA patients have been used previously to study aberrant rRNA processing^7–9,30^. Other features of these cells, such as protein synthesis rates and activation of the p53 pathway have not yet been studied in detail. Here we characterise the phenotype of RP mutated LCLs obtained from DBA patients and suggest that they may be useful for molecular and pharmacological investigations.

## Results

### Characterisation of the pathological phenotype of LCLs from DBA patients

Lymphoblastoid cell lines (LCLs) were established by infection with Epstein-Barr virus (EBV) of primary lymphocytes from 14 healthy subjects and 11 DBA patients. All patients had a heterozygous loss-of-function mutation in *RPS19*, *RPL5*, *RPL11* or *RPL35A* (Table 1).

**Table 1:** Genotype of LCLs established from DBA patients. All mutations are heterozygous.

ID	GENE	MUTATION
**S19-1**	RPS19	c.283_284delG; p.Gly95Alafs*16
**S19-2**	RPS19	c.36_37insAG; p.Glu13Argfs*17
**S19-3**	RPS19	c.280 C > T; p.Arg94*
**S19-4**	RPS19	c.341delA; p.Lys115Argfs*9
**S19-5**	RPS19	c.58delG; p.Ala20Profs*9
**L5-1**	RPL5	c.147 C > G; p.Tyr49*
**L5-2**	RPL5	c.132 C > A; p.Tyr44*
**L5-3**	RPL5	c.1 A > G; p.Met1?
**L11-1**	RPL11	c.451_458delATTGGGGC; p.Ile151Glnfs*18
**L11-2**	RPL11	c.94_95dupA; p.Leu33Thrfs*22
**L35A**	RPL35A	deletion of the whole gene

**Figure 1:**
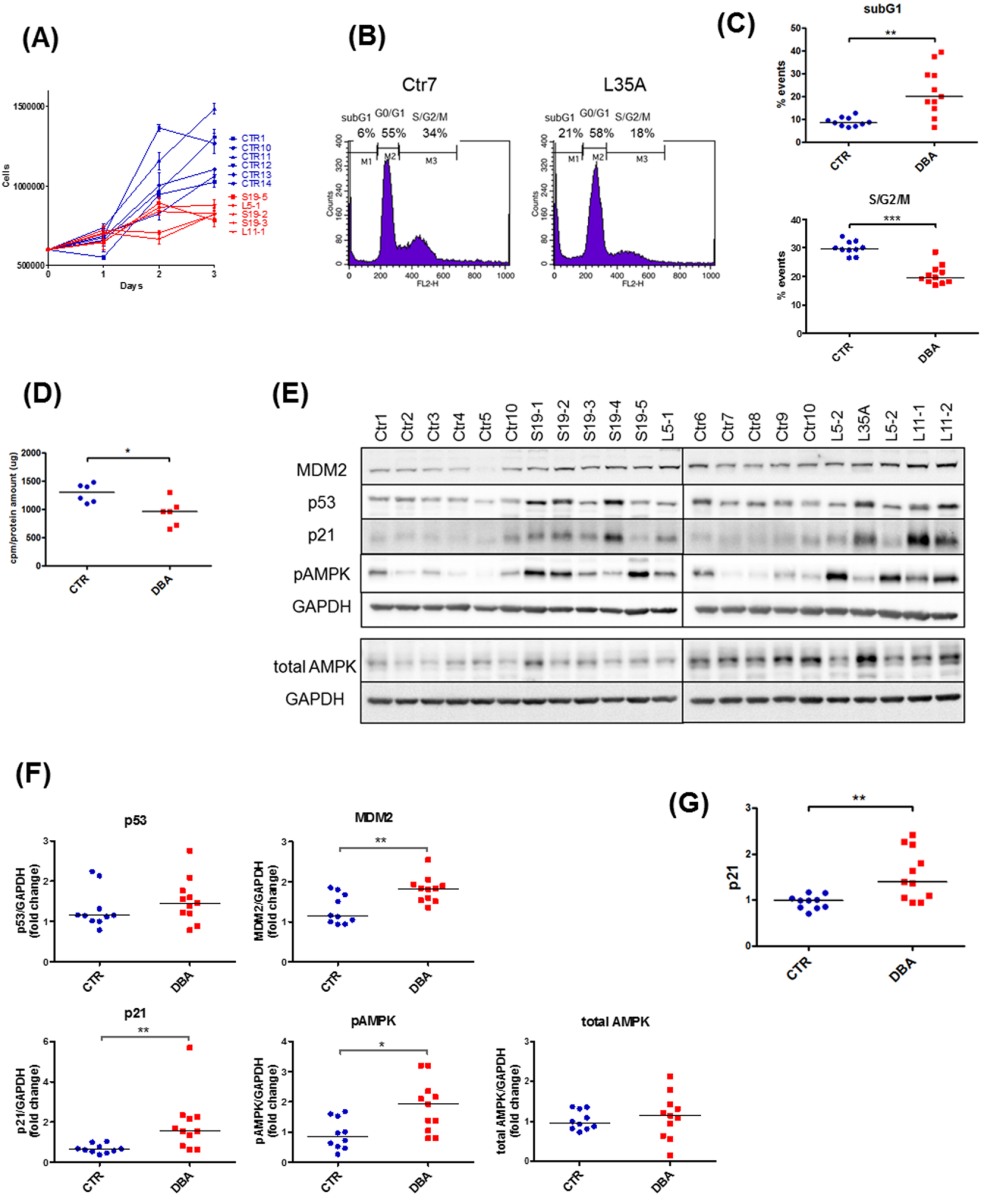
Characterisation of the pathological phenotype of LCLs from DBA patients. (**A**) Proliferation curve obtained by cell count. Growth rate in DBA LCLs was significantly lower than in controls after three days (p = 0.0043). Blue: controls, red: DBA patients. (**B**) Representative cell cycles of control and DBA cells; cells were fixed, stained with propidium iodide and subjected to flow cytometry. M1: subG1; M2: G0/G1; M3: S/G2/M. (**C**) Box plots reporting the mean percentages of cells in subG1 and S/G2/M phases. DBA patients show a higher proportion of cells in subG1 and a lower proportion of cells in S/G2/M phase, compared to controls. Ten control and 11 DBA LCLs were examined. Horizontal line represents median. **p value ≤ 0.01, ***p value ≤ 0.001. (**D**) General protein synthesis is decreased in DBA cells, as assessed by incorporation of [^35^S]methionine/cysteine. Horizontal line represents median. *p value ≤ 0.05. (**E**) Western blot shows an increase in the levels of p53, p21, MDM2 and phosphoAMPK (Thr172), whereas the level of total AMPK is unchanged. (**F**) Densitometric analysis on western blot data from three separate experiments. Horizontal line represents median. (**G**) Expression of p21 transcript, measured by qRT-PCR, is increased in DBA patients. Horizontal line represents median.

DBA cells had a significantly lower growth rate compared to LCLs from healthy controls, as determined by cell counting on day 3 after seeding (Fig. 1A) and a reduced proportion of cells in S/G2/M phase (Fig. 1B,C). Late-stage apoptotic and necrotic cells, that appear in the subG1 region, were increased in number in DBA LCLs (Fig. 1C). General protein synthesis, as measured by incorporation of [^35^S]methionine/cysteine into newly synthesised proteins, was decreased (Fig. 1D), as expected in cells with a reduced number of functional ribosomes. Ribosomal stress is known to activate the tumour suppressor p53 pathway^20,22^, and, accordingly, p53 protein level was generally increased in patients’ cells compared to controls (Fig. 1E,F). In addition, both transcript and protein levels of p21, a target of p53, were increased (Fig. 1E–G). To search for potential biomarkers for the disease, we analysed the expression of AMP-activated protein kinase (AMPK), a protein involved in ribosomal stress^31^, and MDM2, an important negative regulator of p53^32^. Several DBA LCLs showed an increase in the phosphorylation of Thr172, a key activating site in AMPK, when compared with LCLs from normal controls, whereas the total expression of AMPK was not altered (Fig. 1E,F). MDM2 levels were also increased (Fig. 1E,F) consistent with higher p53 levels and subsequent MDM2 transcriptional activation^32^.

### The phenotype due to RP mutations can be rescued by gene therapy

**Figure 2:**
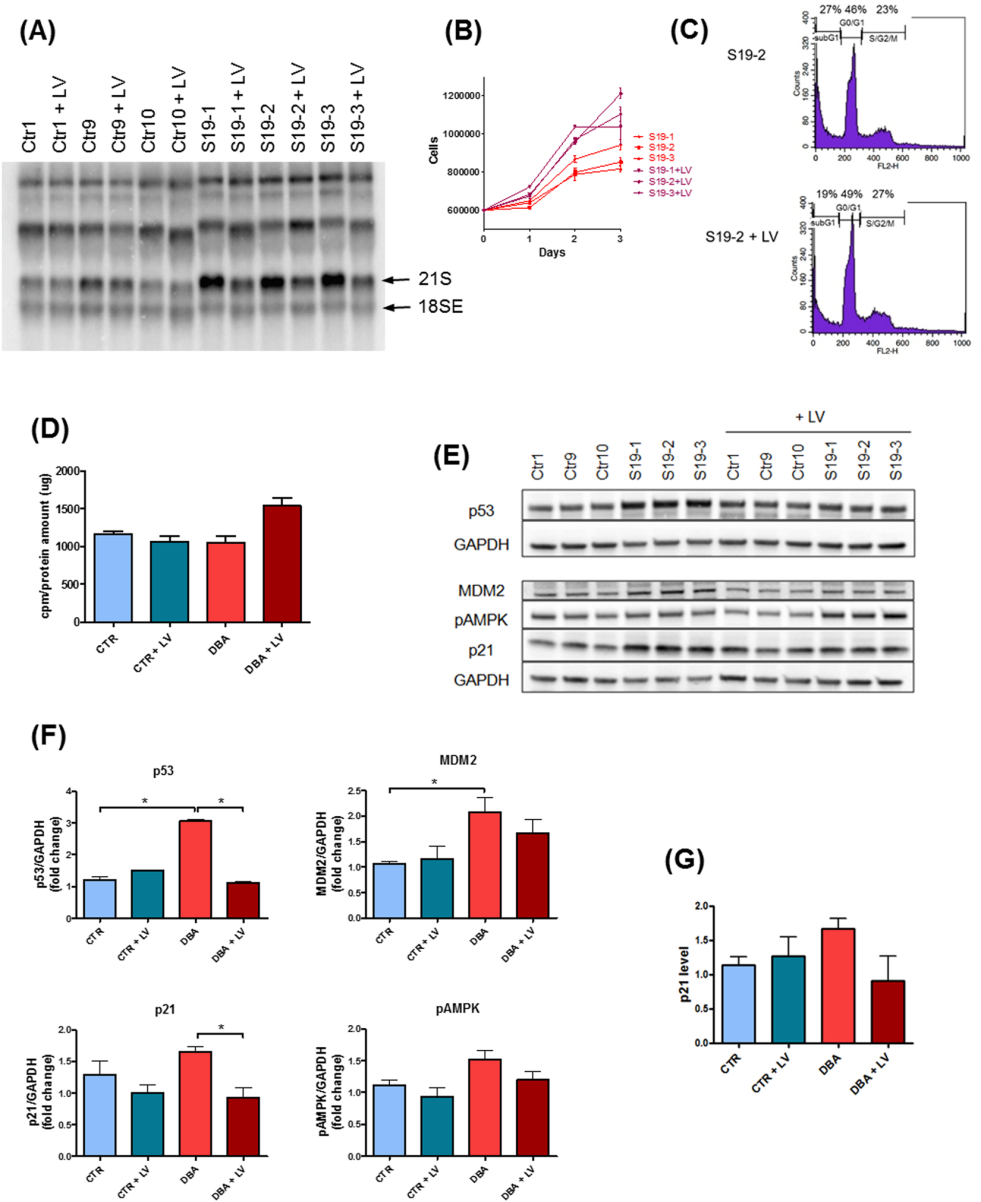
The phenotype due to RPS19 mutations can be rescued by the expression of a RPS19 transgene. (**A**) Northern blot: normal maturation of rRNA is restored in RPS19 mutated patients after expression of an exogenous RPS19 cDNA. The probe labels 18S precursors. (**B**) Proliferation curve: the expression of RPS19 cDNA rescues the growth defect in *RPS19*-mutated patients. (**C**) Representative cell cycles in S19–2 LCL after transduction with LV-RPS19: percentage of subG1 cells was decreased and percentage of S/G2/M cells was increased. LV:LV-RPS19. (**D**) Protein synthesis, measured by incorporation of [^35^S]methionine/cysteine, is improved in patient cells after transduction with LV-RPS19. (**E**) Western blot shows a normalisation in the levels of p53, p21, MDM2 and phospho-AMPK. (**F**) Densitometric data calculated on three western blot experiments. (**G**) Level of p21 transcript in control and *RPS19*-mutated LCLs: transduction with LV-RPS19 reduced p21 in DBA patients.

**Figure 3:**
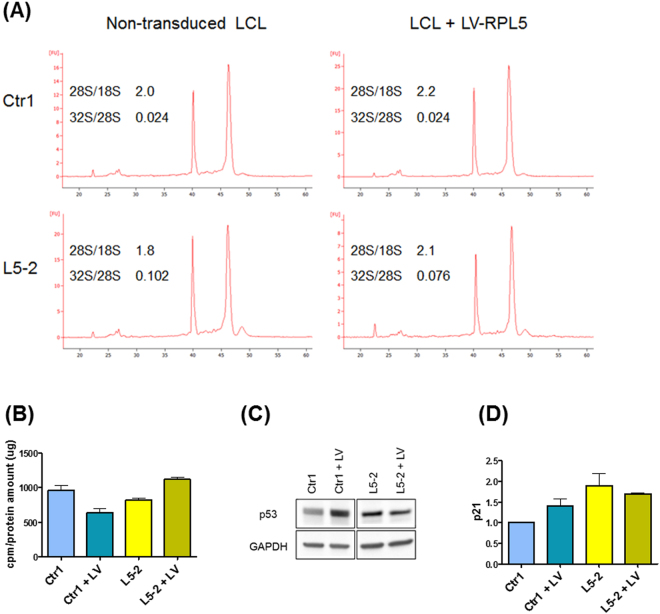
A RPL5 transgene ameliorates the pathological phenotype of a *RPL5*-mutated LCL. (**A**) Deficient rRNA maturation in *RPL5*-mutated cells is partially rescued by transduction with LV-RPL5. Normal 28S/18S ratio ranges between 1.9 and 2.2 in our experimental model. *RPL5*-mutated cells have a reduced 28S/18S ratio due to deficient 28S maturation, together with a prominent 32S pre-RNA peak. Expression of RPL5 after transduction with LV-RPL5 can improve the production of mature 28S rRNA. (**B**) Protein synthesis, measured by incorporation of [^35^S]methionine/cysteine, is improved after transduction with LV-RPL5 in patient cells, but decreased in control cells. LV:LV-RPL5. (**C**) p53 level is slightly decreased in *RPL5-*mutated cells after RPL5 gene transfer, but increased in control cells. LV:LV-RPL5. Original uncropped blots are shown in Supplementary Information. (**D**) Quantitative RT-PCR showing expression of p21:transduction with LV-RPL5 increases p21 expression in control cells, and slightly decreases p21 level in *RPL5-*mutated cells. LV:LV-RPL5.

We then asked whether the phenotypic alterations caused by *RPS19* mutations could be recovered by gene complementation. We transduced three control LCLs and three *RPS19*-mutated LCLs with a lentiviral vector carrying RPS19 cDNA (LV-RPS19, Suppl. Fig. S1). GFP was used as a reporter gene to monitor transduction efficiency by flow cytometry. GFP^+^ cells that ranged between 44% and 76% of total cells (data not shown) were sorted to obtain a pure population (Suppl. Fig. S2). The exogenous RPS19 increased the amount of RPS19 mRNA in DBA patients and, to a lesser extent, in healthy controls; the average fold change in patients was 1.5 (Suppl. Fig. S3). *RPS19*-mutated LCLs show a defective maturation of pre-rRNAs resulting in the accumulation of 21S pre-rRNA (Fig. 2A), in accordance with previous observations in other RPS19-depleted cells^19,33^. Pre-rRNA processing was fully restored by the expression of the RPS19 transgene (Fig. 2A). All the other parameters we examined were partially improved or fully rescued by the expression of the transgene (Fig. 2B–G). In particular, proliferation and protein synthesis were increased (Fig. 2B–D), whereas the expression of p53 and p21 was significantly reduced (Fig. 2E–G). These results show that pathogenic phenotypes observed in patient LCLs are specifically due to *RPS19* haploinsufficiency and can be recovered by the expression of a RPS19 transgene. We performed a similar experiment on *RPL5*-mutated LCL transduced with a vector expressing RPL5 cDNA (LV-RPL5). We found that the expression of the RPL5 transgene achieved a partial rescue of 28S rRNA maturation in the patient LCL, as shown by the reduction of the 32S rRNA precursor, that accumulates in *RPL5*-deficient cells (Fig. 3A). Protein synthesis rate was restored to normal values (Fig. 3B) and the levels of p53 and p21 were slightly diminished (Fig. 3C,D). Notably, the overexpression of RPL5 seemed to have a deleterious effect on a control LCL, reducing general protein synthesis and inducing p53 and p21 upregulation (Fig. 3C,D).

## Discussion

Although anaemia is the main clinical symptom of DBA, the erythroid lineage is not the only tissue affected by RP mutations, as widely demonstrated by studies on lymphocytes and fibroblasts isolated from DBA patients^12,33,34^. More than 200 different causal mutations have been identified in DBA patients so far^18^. Given the current lack of a simple model system that recapitulates the disease hallmarks we were prompted to evaluate the phenotype of LCLs from patients with known mutations in RP genes.

**Figure 4:**
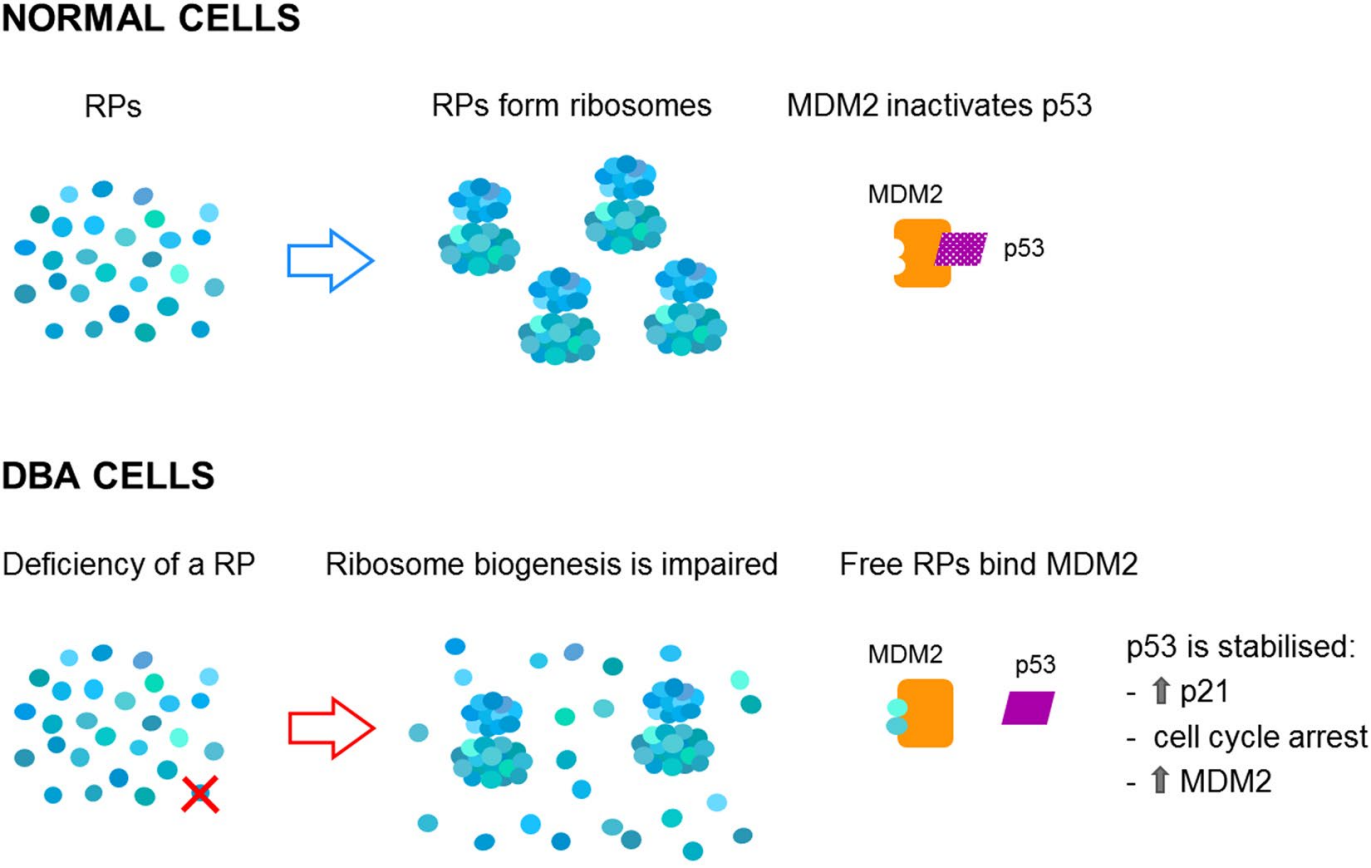
Scheme depicting the interplay among p53, MDM2 and RPs. In normal cells, MDM2 targets p53 for degradation by the proteasome. In DBA cells, the deficiency of a RP perturbs ribosome assembly and causes the accumulation of ribosome-free RPs, such as RPL5 and RPL11, that bind MDM2 and inhibit its activity on p53. Once stabilised, p53 can activate its downstream effectors.

Establishment of LCLs is straightforward and it only requires a small amount of peripheral blood and so useful for specimens from young children. Contrary to other immortalised cell lines, LCLs retain normal p53 function^35,36^. Here we show that RP deficient LCLs exhibit many of the characteristics of primary haematopoietic cells from DBA patients. These characteristics include defective rRNA processing, reduced proliferation, decreased protein synthesis, and activation of p53 and its target p21. In addition to an increased level of pAMPK, which has been previously reported in a prostate carcinoma cell line transfected with RPS19-specific small interfering RNAs^31^, we observed an increase in MDM2 expression. MDM2 is a ubiquitin E3 ligase that targets p53 for degradation by the proteasome. The binding of different RPs, especially RPL5 and RPL11, to MDM2, inhibits its activity leading to stabilisation of p53^20^, that, in turn, induces MDM2 expression^32^. Therefore it is likely that the accumulation of free RPs due to aberrant ribosome biogenesis in RP-deficient cells is responsible for the increased levels of p53 and MDM2^20^ (Fig. 4).

All these pathological features were specifically caused by RP deficiency and could be corrected by gene complementation. Gene transfer in haematopoietic cell lines can be troublesome, especially in cells with a reduced proliferation rate, therefore we used a third generation lentiviral system^37^ and were able to obtain an average transduction efficiency of 59%. To our knowledge, this is the first report that shows normalisation of ribosome biogenesis and function in DBA LCLs. RPS19 gene transfer achieved a rescue of the phenotype in *RPS19*-mutated patients without adverse effects on control LCLs, whereas RPL5 gene transfer appeared to trigger a stress response in control cells, with the upregulation of p53. This is not unexpected, since RPL5 can bind MDM2 and promote p53 stabilisation^20^, and suggests that gene therapy would be more feasible for *RPS19* than for *RPL5-*mutated patients, unless the number of copies of the transgene are precisely quantified to avoid overexpression. However, research on a larger number of subjects is needed to confirm this finding.

The employment of LCLs could be a useful tool to easily explore the effects of many different mutations and RP-specific pathways. Our approach might also be advantageous for diagnostic applications and drug screening. Moreover, our results indicate that, more than an erythroid-specific disorder, DBA should be considered a systemic disease.

## Methods

### Patients and cell cultures

All the studies were conducted in accordance with the Declaration of Helsinki. A written informed consent was signed by all the subjects or their parents or guardians on behalf of the minors/children. The study was approved by the ethics committee at the University Children,s Hospital in Freiburg. After collection of peripheral blood, mononuclear cells were isolated by Ficoll density gradient centrifugation and infected with Epstein-Barr Virus (EBV). Cells were maintained in RPMI 1640 medium supplemented with 10% foetal bovine serum and antibiotics (100 U/ml penicillin and 100 μg/ml streptomycin) and were incubated at 37  °C in a humidified atmosphere with 5% CO_2_.

### Cell proliferation and cell cycle assays

LCLs were seeded in triplicate at a density of 600,000 cells/ml in 25 cm^2^ flask. They were diluted 1:2 with trypan blue dye (purchased from Sigma-Aldrich) and counted, for three consecutive days, in a Neubauer chamber. Cell growth quantification, for each cell line, was carried out using the counting of viable cells. The means of three independent experiments were calculated and plotted in a graph.

For cell cycle analysis LCLs were seeded at a density of 250,000 cells/ml and collected after 48 hours. They were fixed with 70% ethanol, washed with PBS, treated with RNase A, stained with propidium iodide 50 ug/ml, then subjected to flow cytometry analysis.

### Protein labelling

For general protein synthesis analysis, 1 × 10^6^ lymphoblastoid cells were incubated for 30 min with [^35^S]methionine/cysteine (PRO-MIX, GE Healthcare, > 1000 Ci/mmol) to a final concentration of 10 μCi/ml. Cells were lysed in PBS–SDS buffer (150 mM NaCl, 2.7 mM KCl, 8 mM Na_2_HPO_4_, 1.4 mM KH_2_PO_4_ and 0.1% SDS) and proteins were precipitated in 10% (w/v) trichloroacetic acid (TCA). After three washes with 5% (w/v) cold TCA, the insoluble material was collected on GFC filters (Whatman) and the incorporated radioactivity was measured in scintillation counting.

### RNA isolation and analysis

Total RNA extraction was performed using TRIzol Reagent (Invitrogen), followed by on-column DNase treatment and purification with miRNeasy Mini Kit (Qiagen). To study the alterations of rRNA maturation, RNA was either examined by the Agilent 2100 Bioanalyzer System, or subjected to Northern blot. Four μg of total RNA was fractionated on 1.5% formaldehyde agarose gels and transferred to a positively charged nylon membrane (Roche). The RNA was immobilised on the membrane by UV-crosslinking (120 milliJoules/cm^2^). Hybridisation was performed as previously described^19^.

For quantitative RT-PCR, cDNA was synthesised using the High Capacity cDNA Reverse Transcription Kit (Applied Biosystems). Real-time PCR amplification was performed in triplicate using Power SYBR® Green PCR Master Mix (Applied Biosystems) and specific primers (available upon request). *GAPDH* or *ACTB* (β-actin) were used as reference genes.

For Bioanalyzer experiments total RNA was analysed as previously described^34^.

### Western blot analysis

For western blot analysis cells were harvested after 48 hours from the last split and lysed in lysis buffer containing 350 mM NaCl, 1 mM MgCl_2_, 50 mM Tris–HCl (pH 7.5), 0.5 mM EDTA, 0.1 mM EGTA, 1% NP-40, aprotinin 1 mg/ml, phenylmethylsulfonyl fluoride 100 mg/ml and 1% [vol/vol] phosphatase inhibitor cocktail II and III from Sigma-Aldrich. Protein concentration was measured by Bio-Rad Bradford reagent. Protein samples were prepared by addition of Laemmli Sample buffer and resolved on 8–12% SDS-PAGE (Sodium dodecyl sulfate–polyacrylamide gel), transferred onto nitrocellulose Protran membrane (Schleicher & Schuell), and incubated with the following primary antibodies and antisera: mouse monoclonal anti-GAPDH (Millipore, MAB374), rabbit polyclonal anti-MDM2 (Santa Cruz Biotechnology, sc-813), mouse monoclonal anti-p53 (Santa Cruz Biotechnology, sc-126), rabbit polyclonal anti-p21 (Santa Cruz Biotechnology, sc-397), mouse monoclonal anti-AMPK (Santa Cruz Biotechnology, sc-74461), rabbit monoclonal anti-phospho AMPK Thr172 (Cell Signaling Technologies, #2535). Primary antibodies were revealed using horseradish peroxidase-conjugated anti-rabbit or anti-mouse Ab (Jackson Immunoresearch) and the ECL Clarity Western substrate detection (Bio-Rad). Quantification analyses were performed by LAS3000 Image System (Fuji) and ImageQuant software (GE Healthcare). Original uncropped blots are shown in Supplementary Information.

### Vector system for RPS19 and RPL5 rescue

For rescue experiments, we used a third generation lentiviral vector (LV) system. RPS19 or RPL5 cDNAs were cloned and inserted in a bidirectional transfer vector under the control of the phosphoglycerate kinase (PGK) ubiquitous promoter, whereas a minimal human cytomegalovirus (minCMV) promoter drove the expression of the Green Fluorescent Protein (GFP) oriented in the opposite direction to the transgene (Suppl. Fig. S1)^37^. LVs were produced after transient transfection of 293 T cells with the packaging plasmids (pMDLg/pRRE, pRSV-REV and pMD2-VSVG) and the transfer vector for RPS19 or RPL5 (LV-RPS19, LV-RPL5). LCLs were transduced with 10 multiplicity of infection (MOI). Transduction efficiency was monitored by GFP detection; two weeks after transduction, GFP^+^ cells were sorted (Suppl. Fig. S2) using a FACSAria™ III sorter (BD Biosciences) and recultured as described above.

### Statistical analysis

Results are expressed as mean values of technical replicates. Comparison among control and DBA LCLs was made using the two-tailed nonparametric Mann-Whitney test. The efficacy of RPS19 transgene in rescue experiments was evaluated using the one-tailed nonparametric Mann-Whitney test. A p value ≤ 0.05 was considered statistically significant.

## Electronic supplementary material

Supplementary Information
