# Author Correction: Lymphoblastoid cell lines from Diamond Blackfan anaemia patients exhibit a full ribosomal stress phenotype that is rescued by gene therapy

**DOI:** 10.1038/s41598-018-35522-0

**Published:** 2018-11-16

**Authors:** Anna Aspesi, Valentina Monteleone, Marta Betti, Chiara Actis, Giulia Morleo, Marika Sculco, Simonetta Guarrera, Marcin W. Wlodarski, Ugo Ramenghi, Claudio Santoro, Steven R. Ellis, Fabrizio Loreni, Antonia Follenzi, Irma Dianzani

**Affiliations:** 1 Department of Health Sciences, Università del Piemonte Orientale, Novara, Italy; 2 Department of Biology, University of Rome Tor Vergata, Roma, Italy; 3 Department of Medical Sciences, University of Torino, and Human Genetics Foundation (HuGeF), Torino, Italy; 4 Department of Paediatrics and Adolescent Medicine, Division of Paediatric Hematology and Oncology, Medical Center, Faculty of Medicine, University of Freiburg, Freiburg, Germany; 5 Department of Public Health and Paediatric Sciences, University of Torino, Torino, Italy; 6 Department of Biochemistry and Molecular Genetics, University of Louisville, Louisville, KY USA

Correction to: *Scientific Reports* 10.1038/s41598-017-12307-5, published online 20 September 2017

The Acknowledgements section of this Article is incomplete.

“The authors wish to thank the patients and their families for providing biological specimens for this study. We thank Dr. Davide Rossi for assistance with the Bioanalyzer experiments. This work was supported by Telethon grant GGP13177 (to ID and FL), Fondazione Europea per la DBA (to AA), Gruppo di Sostegno DBA Italia (to ID and UR), Banca del Piemonte (to UR).”

should read:

“The authors wish to thank the patients and their families for providing biological specimens for this study. We thank Dr. Davide Rossi for assistance with the Bioanalyzer experiments. This work was supported by Telethon grant GGP13177 (to ID and FL), Fondazione Europea per la DBA (to AA), Gruppo di Sostegno DBA Italia (to ID and UR), Banca del Piemonte (to UR). This work was also supported by E-RARE EuroDBA consortium grant (BMBF #01GM1301 and 01GM1609) to MWW. AF was partially supported by ERC grant #261178.”

