# Supplementary material for: Lymphoblastoid cell lines from Diamond Blackfan anaemia patients exhibit a full ribosomal stress phenotype that is rescued by gene therapy
Source: Sci Rep. 2017 Sep 20;7:12010. doi: 10.1038/s41598-017-12307-5 (PMC5607337; doi:10.1038/s41598-017-12307-5)

Supplementary Information

Lymphoblastoid cell lines from Diamond Blackfan anaemia patients exhibit a full ribosomal stress phenotype that is rescued by gene therapy

Anna Aspesi[1+*], Valentina Monteleone[2+], Marta Betti[1], Chiara Actis[1], Giulia Morleo[1], Marika Sculco[1], Simonetta Guarrera[3], Marcin W. Wlodarski[4], Ugo Ramenghi[5], Claudio Santoro[1], Steven R. Ellis[6], Fabrizio Loreni[2], Antonia Follenzi[1], Irma Dianzani[1]

[1] Department of Health Sciences, Università del Piemonte Orientale, Novara, Italy
[2] Department of Biology, University of Rome Tor Vergata, Roma, Italy
[3] Department of Medical Sciences, University of Torino, and Human Genetics Foundation (HuGeF), Torino, Italy
[4] Department of Paediatrics and Adolescent Medicine, Division of Paediatric Hematology and Oncology, Medical Center, Faculty of Medicine, University of Freiburg, Freiburg, Germany
[5] Department of Public Health and Paediatric Sciences, University of Torino, Torino, Italy
[6] Department of Biochemistry and Molecular Genetics, University of Louisville, Louisville, KY, USA.

[+] These authors contributed equally to this work
* Corresponding author: Anna Aspesi, Department of Health Sciences, Università del Piemonte Orientale, via Solaroli 17, 28100 Novara, Italy. Phone +39 0321 660643, fax +39 0321 620421.
E-mail: anna.aspesi@med.uniupo.it

Supplementary Figure S1. Schematic representation of the LV used to express RPS19 cDNA (LV-RPS19). minCMV: minimal cytomegalovirus promoter.

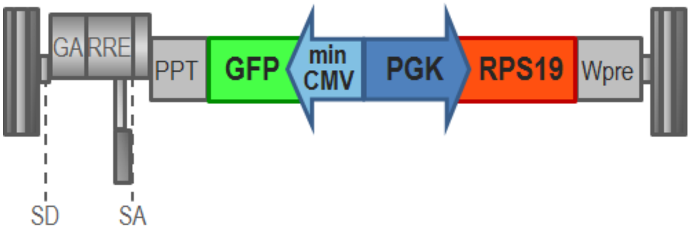

Supplementary Figure S2. Sorting of LCLs transduced with LV-RPS19. Transduction efficiency was measured by flow cytometry and GFP+ cells were sorted. Sample LCL S19-2 is shown.

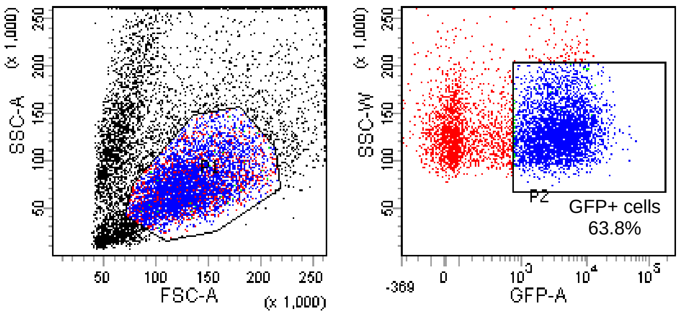

Supplementary Figure S3. RPS19 mRNA expression measured by qRT-PCR. *RPS19*-mutated patients show a lower level of RPS19 transcript, which is increased after LV-RPS19 transduction. Experiments were performed in triplicate on cells established from 3 healthy controls and 3 RPS19-mutated patients. * $p \leq 0.05$

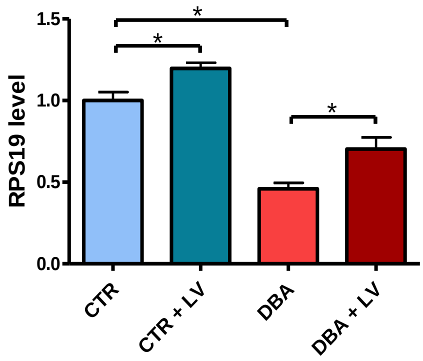

Uncropped blots

Figure 1E

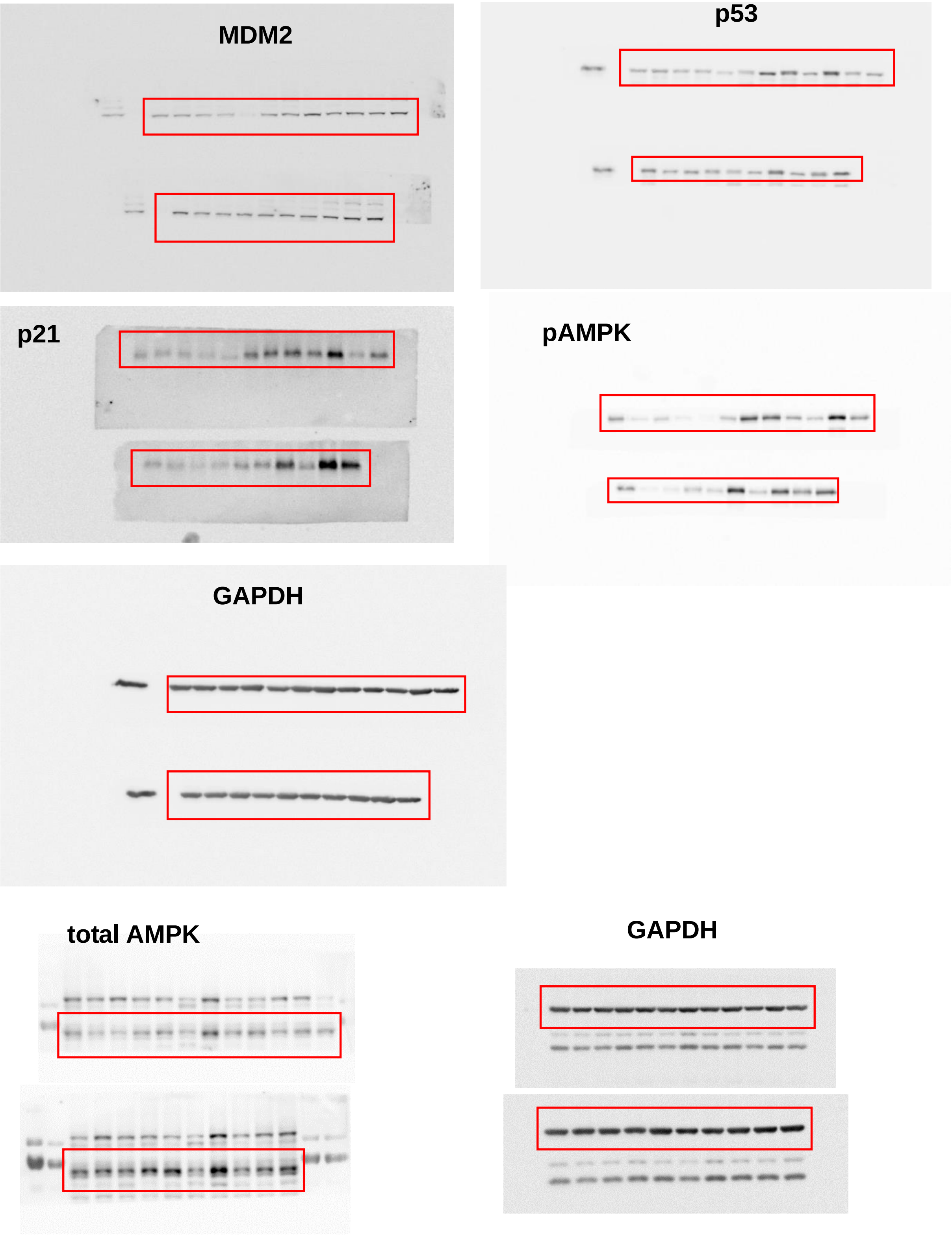

Figure 2A

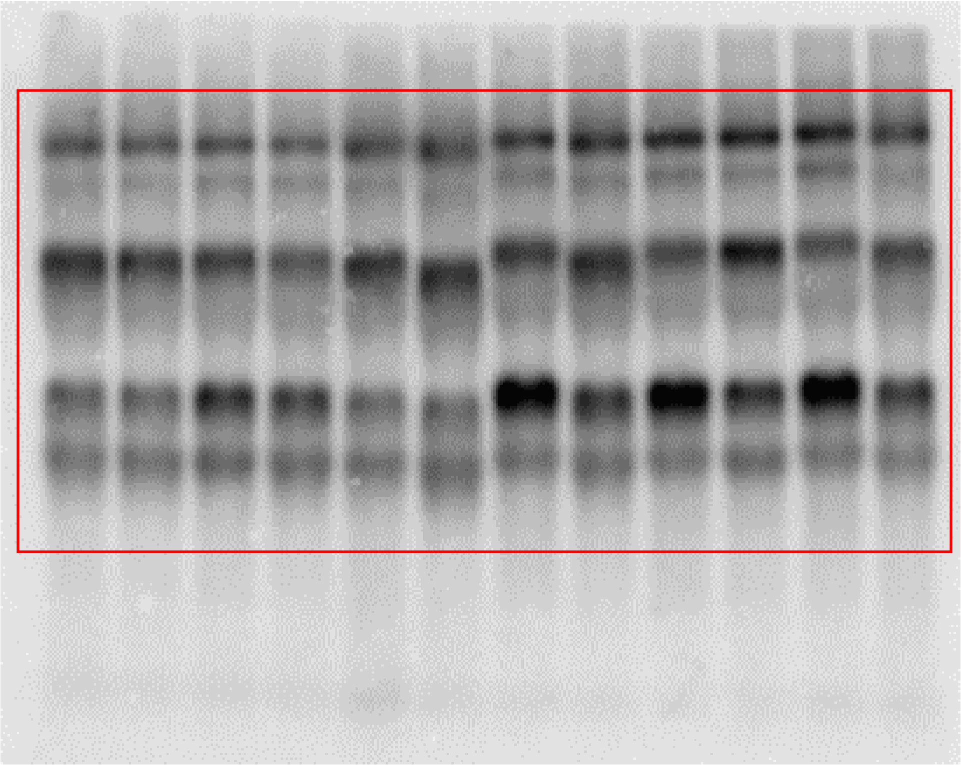

Figure 2E

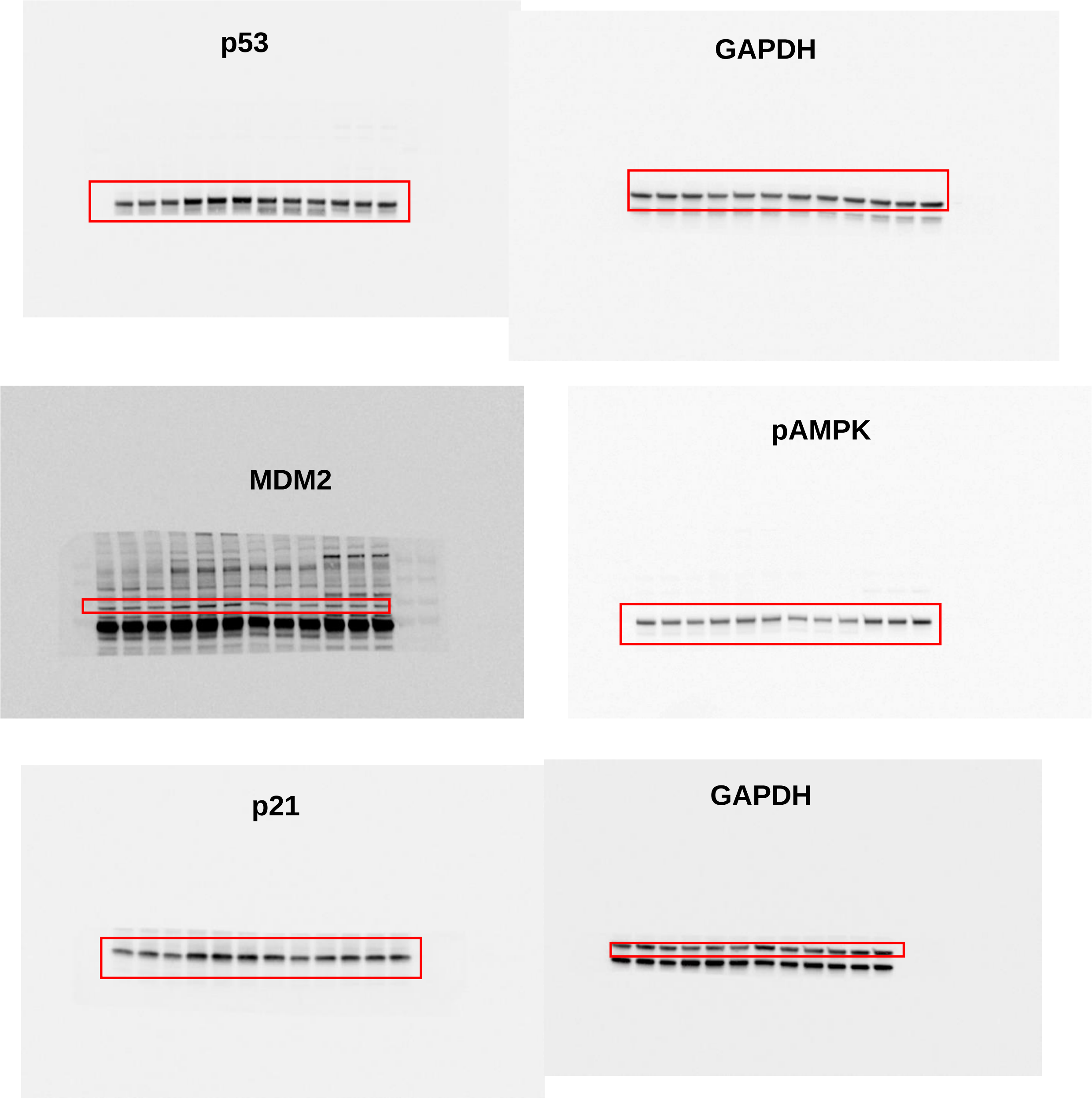

Figure 3C

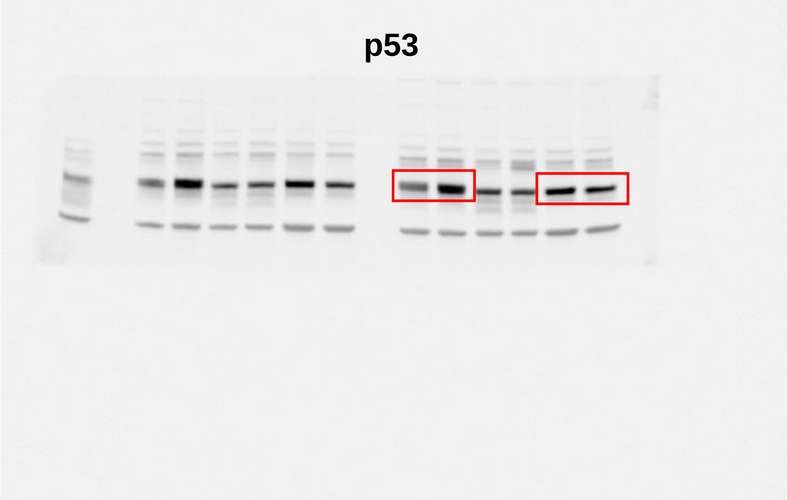

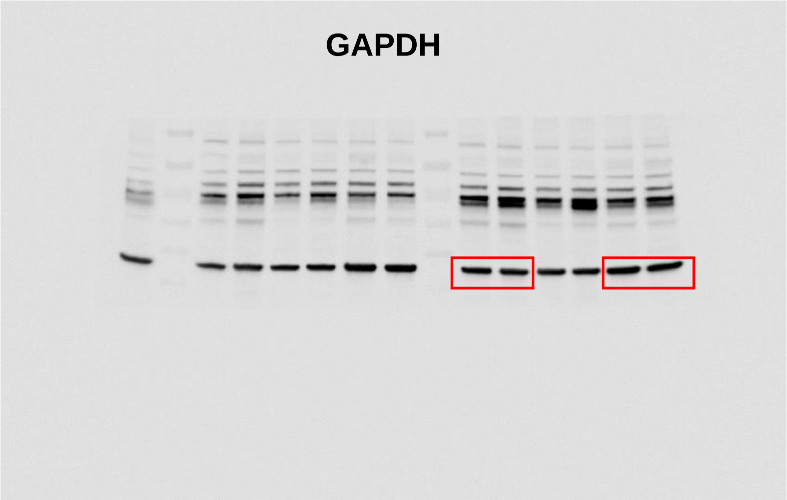

Supplement: Supplementary file 1 — Supplementary Information [file 41598_2017_12307_MOESM1_ESM.pdf]
